# Solving “Smart City” Transport Problems by Designing Carpooling Gamification Schemes with Multi-Agent Systems: The Case of the So-Called “Mordor of Warsaw”

**DOI:** 10.3390/s18010141

**Published:** 2018-01-06

**Authors:** Robert Olszewski, Piotr Pałka, Agnieszka Turek

**Affiliations:** 1 Faculty of Geodesy and Cartography, Warsaw University of Technology, 00-661 Warszawa, Poland; r.olszewski@gik.pw.edu.pl; 2 Faculty of Electronics and Information Technology, Warsaw University of Technology, 00-661 Warszawa, Poland; p.palka@ia.pw.edu.pl

**Keywords:** smart city, gamification, carpooling, multi-agent system, sustainable transport, sustainable city, spatial data mining, IoT, geoinformation

## Abstract

To reduce energy consumption and improve residents’ quality of life, “smart cities” should use not only modern technologies, but also the social innovations of the “Internet of Things” (IoT) era. This article attempts to solve transport problems in a smart city’s office district by utilizing gamification that incentivizes the carpooling system. The goal of the devised system is to significantly reduce the number of cars, and, consequently, to alleviate traffic jams, as well as to curb pollution and energy consumption. A representative sample of the statistical population of people working in one of the biggest office hubs in Poland (the so-called “Mordor of Warsaw”) was surveyed. The collected data were processed using spatial data mining methods, and the results were a set of parameters for the multi-agent system. This approach made it possible to run a series of simulations on a set of 100,000 agents and to select an effective gamification methodology that supports the carpooling process. The implementation of the proposed solutions (a “serious game” variation of urban games) would help to reduce the number of cars by several dozen percent, significantly reduce energy consumption, eliminate traffic jams, and increase the activity of the smart city residents.

## 1. Introduction

The unprecedented scale of recent urban growth has increased the demand for smart city solutions. Today’s cities consume over two-thirds of the world’s energy and are responsible for over 70% of the global greenhouse gas emissions [1]. In a smart city, efforts to reduce energy consumption are focused not only on supporting the development of smart grid systems or prosumers, but also on reducing traffic. Additional benefits include saving costs and time, environmentally friendly transport, carbon emissions reduction, and fuel consumption reduction. Traffic adversely affects the environment and can contribute to a lower quality of life due to, for example, air pollution, excessive noise levels and excessive energy consumption.

When compared with almost any other means of transport, personal transport is extremely unprofitable, mainly because in most cars there are only one or two passengers. For example, the average car occupancy in the United Kingdom is estimated at 1.59 passengers per car and the average is only 1.05 in Germany [2]. Forecasts estimate that the global number of cars will increase to 2.8 billion and global road emissions will have doubled by 2050 [3]. The issue of sustainable transport is becoming one of the key problems facing contemporary planners and researchers [4]. Therefore, it is crucial to seek innovative solutions that will allow for smart cities to realize the potential of information and communication technology (ICT) and geoinformation, as well as the opportunities provided by the increasingly common Internet of Things (IoT). Such an approach is exemplified by carsharing, which has visibly changed the carbon dioxide emission of the average carsharing participant [5].

Carpooling is a system through which users with similar routes can use one car. Its main goal is to match people who commute to work; therefore, carpooling can be an effective method of alleviating traffic jams during rush hours [6].

The contributions of the paper are: (i) a proposal for solving the problem of traffic jams in “Mordor” without any serious infrastructural investments; (ii) a proposal for the use of gamification mechanisms, which utilize the market programming models with complex limitations, to solve the transport problems in the “Mordor of Warsaw”; (iii) a description of implementation of a multi-agent system which uses this methodology; (iv) a real case study dealing with the transport problems of “Mordor”; and, (v) variant tests for this case study.

The layout of this article is as follows: after a short introduction, there is a critical review and analysis of literature on gamification, carpooling and the modelling by means of multi-agent systems. In Section 3, a description of the problem analysed in the subsequent parts of the article is included. Section 4 consists of the research methodology used in the article, including the analysis of the collected surveys, spatial data mining methods, and the mechanisms by means of which simulation of the gamification is carried out, meaning the implemented multi-agent system. This passage also describes the model of an agent who represents an employee working in “Mordor” and analyses her/his characteristics. Section 5 contains the description, analysis, and argumentation of the conducted variant tests. The last section comprises the summary of the presented results and plans for the future. The attachment contains questions used in the survey.

## 2. Related Work

There are numerous examples of solutions that can support carpooling, but they do not have bearing on the general car occupancy. First, people are neither motivated nor encouraged by their local governments or employers to make use of such solutions. Secondly, in most of the existing systems, it is not possible to organize carpooling in an ad hoc manner, which is a problem as people are used to flexible working hours and the immediate availability of their own car for transportation [2].

Studies carried out by Shaheen et al. [7] identified six main casual carpooling success factors: a time savings incentive for drivers; monetary savings for passengers; pickup locations near freeways, residences, parking, or public transit stops; a common dropoff location; reliable public transit for the return trip; and, a HOV requirement of three or more occupants. Other researches [8,9] show that flexibility is identified as one of the main motivation for casual carpooling.

Kelley [10] introduced a term “enhanced casual carpooling”, referring to the casual carpooling with the incorporation of modern technology. At present, several organized carpooling projects have been proposed, but formalized system on full-scale does not exists today [7].

Berlingerio et al. [11] present a data-driven methodology for GReen And sociAL carpooling (GRAAL). Authors try to optimize a carpooling system by minimizing the number of cars, and, at the same time, by maximizing the enjoyability of people sharing a trip. The enjoyability is measured based on people’s interests, social links, and tendency to connect to people with similar or dissimilar interests. GRAAL computes the enjoyability within a set of users from crowd-sourced data (starting from Twitter data), and then uses it on real world datasets to optimize a weighted linear combination of number of cars and enjoyability.

Elbery et al. [12] is using a geo-social data to recommend individuals to join their friends during trips by using home location models and users’ similarities. Cici et al. [13] derive data using 3G Call Description Records and Twitter. Home and work locations, and social ties between the users are used to develop an algorithm for matching users with similar mobility patterns. Bicocchi and Mamei [14] developed an application for ride sharing by exploiting a clustering algorithm that is applied to labeled trajectories. Byon et al. [15] develops Facebook-based carpooling, and proposes Twitter-based traffic monitoring and Flickr-based incident reporting applications. Zahng et al. [16] propose a carpool service (coRide) in a large-scale taxicab network by analyzing the reduction of circulating taxis in presence of ride sharing. Dispatching cloud servers calculate cost-efficient carpool routes for taxicab drivers, and thus lower fares for the individual passengers. Knapen et al. [17] introduce a system (GCPMS—Global CarPooling Matching Service) that provides carpooling advises by maximizing the expected value for negotiation success.

Fahnenschreiber et al. [18] presented a solution that integrates dynamic ride-sharing into existing multi-criteria intermodal travel information system. The authors allow for dynamic ride-sharing between two train rides by connecting public transport stations by dynamic ride-sharing offers of drivers. Then, the system finds a suitable dynamic ride-sharing offers of drivers, who can take a passenger from start location to a public transport station of from the station to the needed location.

Car pooling can be operated in two main ways: Daily Car Pooling Problem (DCPP, where users must agree on how to carpool on a daily basis) or Long-term Car Pooling Problem (LCPP, where users form groups and organize shared rides that persist during a certain period of time) [19,20].

In Bruck et al. [19] authors tried to solve a daily carpooling problem with the aim of reducing CO_2_ emissions by creating a web application prototype using two mathematical formulations and two heuristic algorithms. Daily carpooling problem was also analyzed by Wolfler Calvo et al. [20], who developed an integrated system to organize and manage carpooling groups. The core of the system is an optimization module that solves heuristically the specific routing problem and operates as a two-step procedure.

Yan et al. [21] deals with the long-term car pooling problem. The developed model is formulated as a special integer multiple-commodity network flow problem, and a Lagrangian relaxation-based algorithm is developed to solve the problem. Naoum-Sawaya et al. [22] were also analyzing a LCPP. They proposed a stochastic optimization model for the optimal placement of vehicles for ridesharing systems.

Montes et al. [23] developed an online community for collaborative consumption centered in the Senegalese community that travels by car from Europe to Africa—Teranga Go! (http://terangago.com). Carpooling relationships are based on the sense of a real existing community, social experiences among users, and connection through technology, where confidence is the key concept. The authors applied Multi-Expert Multi-Criteria Decision Making model using Hesitant Fuzzy Linguistic Terms to represent the expert opinions.

Globally, there are examples of commercial applications that explore the concept of carpooling; the journeys that are assumed by most of these are one-off long trips, although they do not exclude shorter repeated ones. The most popular commercial applications are: Blablacar [24], the biggest carpooling solution in Poland and the rest of Europe; Amovens [25]; compartir [26]; and, ZimRide [27], which is the largest carpooling solution in the United States. Caballero-Gil et al. [28] describes a trust-based cooperative social system that is realized as a mobile application. The author discusses other interpretations of carpooling, and proposes the trust-based one: a combination of reputation and privacy protection. Bonhomme et al. [29] describes a multi-agent platform, in which emphasis is put on the security services that allow for the mutual authentication of the commuters. Megalingam et al. [30] describe the Automated Wireless Carpooling System, discussing the safety and security of passengers.

Ronald et al. [31] proposed an agent-based model that focuses on the negotiation methodology. The described model includes a well-defined and structured interaction protocol, integrating the transport and social layer. A utility function is presented on the basis of individual and combined attributes. The agents negotiate on the type, location, and the start time of social activity.

Nourinejad and Roorda [32] formulated centralized (binary integer programming) and decentralized (dynamic auction-based multi-agent) optimization algorithms to match passengers and drivers.

Galland [33] briefly describes a conceptual design of the carpooling application that was initially proposed by Cho et al. [34], Bellemans et al. [35], and Galland et al. [36]. This design uses an agent-based model on the Janus multi-agent platform [37]. This platform permits individuals to select the best transport mode for their characteristics, to maintain a social network, to negotiate for carpooling, and to carpool the driver and the passengers of a car.

Hussain [38] analyses an organizational-based model for long-term carpooling. The authors claim that to begin the process of carpooling, the objectives of the individuals and their intentions should match. Moreover, they emphasize that success depends on time preferences, route optimization, and on the effect of constraining activities. They applied a multi-agent system, using a social network based on the operational activity-based model for Flanders in Belgium. From the results of the simulation, ran on the Janus multi-agent-based platform [37], they concluded that the simulation time is unacceptable for more than 40,960 agents. They also presented a detailed model of the time and spatial constraints.

An initial prototype of the Dynamic Carpooling System for people who usually share the same route is implemented in NetLogo [39].

Kothari [40] describes the development of the Genghiz multi-agent system for carpooling. The system is developed in the Java Agent Development (JADE) multi-agent framework.

One-way carsharing systems, which allow for their users to rent a car and leave it in any other station, are also being developed. Alfian et al. [5] concentrate on solutions that are connected with forecasting relocation to solve car distribution imbalances for one-way carsharing services.

Some of the other services include Alternetrides [41], ShareYourRide [42], and Ridesharing [43]. Different features are offered by different systems: some offer very basic, simple functions, such as sending requests for lifts in a specific place or time and then looking for suitable co-passengers, depending on demand. Other systems integrate geographic information systems (GIS), providing the locations of passengers and cars on a map. More advanced systems, such as BlueNet-Ride [44], make use of cloud-based architecture to provide easier access to information on vehicles and passengers in real time [6].

Gamification is currently used in several popular applications. Waze app is a social community-based traffic and navigation application. It informs the users about the traffic, construction, accidents, the police, helps to find the cheapest gas along the route, and—in case of a traffic jam—it helps to change the route to save the time, etc. The application encouraged users to help build its crowdsourced maps. It offered points, for example, for driving around with Waze and validating the application’s directions. From time to time, the application provides various games, in which the drivers collect items while driving. The user can also get extra points, for example for the resolution of 50 map errors, notification about road problems, etc. The points reveal more avatars available for users [45].

Another application that influences personal travel behavior decisions is Metropia. It is an advanced platform that allows for travelers to discover mobility possibilities to optimize their travel. Metropia uses mobile technology, real-time data, and back-end predictive algorithms to predict traffic and provide the best route. Behavioral economics strategies and gamification elements are used to incentivize and reward drivers to shift their travel to off-peak times, take under-utilized corridors, and opt for alternative modes of transportation. It encourages the drivers to earn rewards, save time and fuel, or to cut CO_2_ emissions and to contribute to reforestation program [46].

Other platforms that use geoinformation technologies and gamification techniques in transforming urban transportation systems are: commuter management software RideAmigos [47]; TravelWise Tracker [48]—a website which helps people to combat the pollution by energy conserving trips like carpooling, walking, and biking.

Carpooling is not currently popular in Poland, but there are some services dedicated to seeking shared rides. These services are not commonly used and they only provide basic functions, for example, JedziemyRazem [49], or the more advanced mobile application dedicated to connecting commuters, called InOneCar [50]. However, the first carsharing services are starting to appear in Poland: for example, self-operating car renting systems, such as Panek [51] or Traficar [52].

The existing carpooling and carsharing services are “standard”: their rules of operation are very simple, and most applications or services work similarly. However, effective implementation of such solutions—that is, systems that bring measurable results reducing energy consumption or solving transport problems—requires significant innovations regarding both the technology used and social innovations. This is particularly important as far as shaping a geoinformation society in a smart city is concerned. This “non-standard” and unconventional approach may also include using gamification techniques in carpooling, which can be done effectively in the era of IoT, ICT, and geoinformation technologies.

There are many different examples of application of gamification techniques. Ro et al. [53] tested gamification to reduce people’s household electricity consumption by changing their environmental behaviors. Coombes and Jones [54] used gamification to get children more active by encouraging them to walk and cycle (active traveling) to school. Korn and Schmidt [55] consider many different approaches to gamification, among others the use of gamification in service and production. Shreenath et al. [56] investigated the feasibility of implementing gamification in the Swedish transport administration (Trafikverket).

It is assumed that a suitably adopted gamification scheme can contribute to solving transport problems, but this would not replace the infrastructural investments from the city’s authorities. What gamification emphasizes is the role of a human in the process. Behind the idea of gamification—that is, using mechanisms known from games to change human behaviour outside of the game—lies the belief that it is effective only when users join the program voluntarily. Scoring employees for achieving their goals may be a strong incentive that encourages changes in behaviours and habits [57].

## 3. Research Area—Description of the Problems

**Figure 1:**
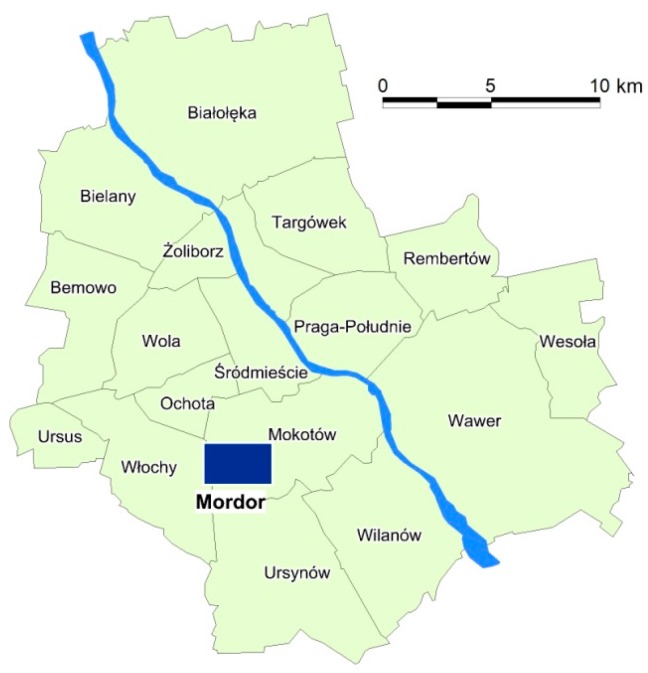
The location of the research area (the Varsovian “Mordor”) within the district division of Warsaw.

The research area is restricted to approximately 6 km^2^ in Służewiec, part of the Mokotów district of Warsaw, on the left bank of the Vistula river, in the south-western part of the city (Figure 1). With ulica Domaniewska (Domaniewska street) at its centre, this is now the biggest office district of Warsaw. According to various estimations, 83,000–100,000 people commute here daily to work in almost 100 buildings that cover over 1 million square meters of floor space [58]. It is the largest concentration of corporations in Warsaw, comprising global and national companies from various industries, as well as trade and services. When most employees choose to commute by car, a true transport apocalypse takes place. In terms of transport, this area is one of the most poorly planned places in the city. During rush hours, people are spilling out of tram stops and it takes an hour to travel the busiest 100 m of the route. All of this is why the residents of Warsaw have nicknamed the area surrounding Domaniewska “Mordor”, a reference to the place where evil forces accumulated in J.R.R. Tolkien’s *Lord of the Rings* trilogy.

The development of this area has been spontaneous, without any spatial management plans. Instead of a coherent urban design, it has been fully subordinated to commercial and market needs. The area’s main problems are: traffic jams; insufficient parking; spatial chaos; and, scarce urban greenery. There are no attractive public spaces, footways, or multifunctional developments. This area is characterized by the predominance of its office-related function, which adversely impacts its aesthetics.

**Figure 2:**
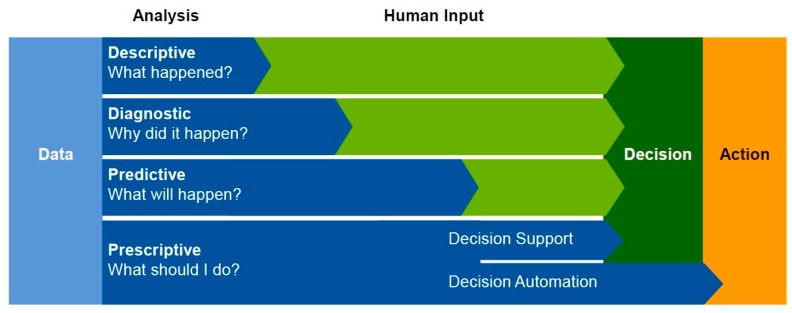
From data to a decision support system (based on [61]).

Research conducted by Biuro Drogownictwa i Komunikacji (the Road Engineering and Transportation Authority) and the #LepszySłużewiec (#BetterSłużewiec) association of employees, tenants, real estate owners and managers, and the residents of Służewiec who want to improve working and living conditions in this area, found that, on average, 36% of office employees commute every day using their own cars (equating to about 40,000 cars, Figure 2). This number does not include workers from industrial plants and older office buildings, or people who travel to Służewiec for a short time, for example, to shop in the nearby shopping centre, Galeria Mokotów [59].

As the analyses conducted by Jones Lang LaSalle (JLL) [60] show, as many as 87% of passenger cars entering the office area of Służewiec during the morning rush hours are occupied by only the driver [59]. As Jan Zombirt says, “the city should initiate, coordinate and champion initiatives aimed at improving the transport and parking situation, supporting, e.g., carpooling, by providing cars that have more than one person with preferential spaces. An application that matches drivers and passengers who travel in the same directions would be a good solution. Also, implementing an intelligent system of identifying parking spaces. Developers could lease their unused parking lots, while employers should think about introducing flexitime that would stretch the rush hours in time” [59].

## 4. Research Methodology

To solve the transport problems of the so-called “Mordor of Warsaw”, it is crucial to galvanize employees by tapping into the potentials of gamification and the common usage of geoinformation technologies, ICT and IoT. At the core of this approach is developing an attractive mobile game that utilizes multi-agent systems, geographical position (GP) navigation, and other information technologies, for example, elements of augmented reality, to make the solution more attractive. Devising a methodology required the sequential implementation of several components:

1. Surveying a representative random sample of people who work in the “Mordor of Warsaw” to identify the preferences, needs, and capabilities of the statistical population of “Mordor”.
2. Analysis using spatial data mining algorithms to identify the key problems and traits that characterize age groups, residents of different city districts, etc.
3. Devising a simulation of changes in the way the “Mordor” employees commute by using variations of gamification, a variable parameterization of the multi-agent system, and optimization of the system.
4. Developing a prototype of a “serious game” for the Varsovian “Mordor”, the methodology of gamification, and an appropriate employee incentives system (financial, point system, ranking, etc.). Basic difference between serious and purely entertainment games is that serious games attempt to achieve specific learning goals [62]. In general, they are designed to attractively and efficiently educate and promote changes in the behavior of participants. Users interact with each other, have fun, and enjoy the game, while gaining awareness and knowledge of the problem to be solved [63].

In this article, numbers 1, 2, and 3 have been discussed as the elements of the process that served as the methodical starting point for optimizing employees’ commutes and solving the problem of traffic jams in a smart city, as exemplified by the “Mordor of Warsaw”. The description of the game, its pilot implementation, and the results obtained will be the subject of a separate publication.

What should be emphasized is that the aim of the research was not simply to solve an individual transport problem of a specific district, but to devise a methodical starting point for a decision support system that would optimize public transport for any city, based on gamification. Analysing the data collected from a representative sample of the population by means of data mining algorithms (or, more specifically, spatial data mining) facilitates a proper system parameterization for any city that aspires to become a “smart city”.

Spatial data mining techniques were used because these techniques enable the detection of previously unknown regularities and relationships, anomalies and trends, and patterns and structures “hidden” in a set of observations. The aim of data mining is to transform “raw” data into useful information and knowledge. Data mining is the process of studying and analysing large amounts of data by means of specific algorithms to detect significant patterns and rules [64,65]. Thus, data contained in databases can be used in decision support systems (Figure 2). The following questions: What happened? Why did it happen? What will happen? What should we do? Can be answered using the respective approaches: descriptive, diagnostic, predictive and prescriptive. Such approaches facilitate the creation of expert systems that support decision processes [65,66,67,68]. The collected data was treated as a representative sample of the statistical population. As a result of the data analysis, a carpooling model (and its incentive system) that uses the process of gamification was devised.

### 4.1. Analysis of Survey Data

The value and potential of a devised system are affected by the data collected and how the data are transformed into useful information and knowledge. The behaviour of a population of people who work in the so-called “Mordor” area was investigated. It was not possible to study the entire population; therefore, a survey to study a representative sample of the population was created. Based on its results, the distribution of population characteristics was inferred. The results were a set of parameters for the multi-agent system.

The survey (Appendix A) was made available via social media (Facebook fan page: “Mordor na Domaniewskiej”, which has over 133,000 users), and a link to the survey was distributed directly by email.

The survey was made available for one week (28 April 2017 to 5 May 2017). There were 337 individual responses given by 180 men (53%) and 157 women (47%). A clear majority of responders (64%) were young people (26–35 years old) at the beginning of their professional careers. Respondents were asked what means of transport they used to commute: 45% use cars (11% company cars); 47% use public transport (bus, tram, subway, railway); and, the rest use bicycles or walk. About 70% of respondents travel directly from home to work, the rest drop their children or family members off at the kindergarten/school/work on the way. On the return journey, 44% of the respondents go directly home, while 56% pick up their relatives and/or go shopping.

The survey was designed in such a way as to gather data related to the amount of time spent travelling to work, the place of work, the transportation method and the directions in which they travel to and from work. Respondents also answered questions about their potential use of carpooling to commute and what type of incentive system they would prefer to encourage sharing private vehicles or to encourage joining somebody in their commute. The second part of the survey consisted of basic information gathering: gender, age, occupation, and the place of residence.

The problems of traffic jams and lengthy journeys to work are important to a clear majority of respondents (79%). Two-thirds of the respondents have never picked up another commuter and have never been invited to commute in somebody else’s car. However, an overwhelming majority of respondents are willing to try carpooling, if properly incentivized. The results indicate that only 16% of women and 24% of men are not willing, under any circumstances, to use carpooling. One can presume that, as the results show, the carpooling solution should be targeted at younger people. Only 17% of respondents under 35 are not willing to try carpooling, while the proportion of older unwilling respondents is 32%.

The results are extremely promising and it may be assumed that it is possible to devise a decision support system optimizing the process of the shared commute to and from work. The requisite conditions are:

1. In terms of the gamification rules and a rewards and incentives system, devising an attractive mobile-based tool in the form of a so-called “serious game” (the rules of the game, which uses the narrative, storytelling and fantastical atmosphere of Tolkien’s Mordor, incentive systems, and scoring will be addressed in a separate article).
2. A system parameterization that uses the characteristics of the statistical population of the employees from the “Mordor of Warsaw”, obtained from the survey results. In a decision support system, it is necessary to consider:
  - Differentiated incentive systems adapted for age, gender, place of residence, incentive preferences, etc.
  - The typical start and finish times of work, based on the generated start and finish work parameters.
  - The elasticity of the start and finish times of work, using information generated from the distribution of start, and finish times.
  - The district in which the person lives.
  - Whether the person must visit another location before and/or after work (kindergarten, shopping, etc.), or must pick up someone, and the person’s willingness to travel together: using this information, the capacities of the cars were generated.
  - From the level of dissatisfaction with the traffic jam problem, the base price was generated: it is assumed that to get rid of the problem a more dissatisfied person wants to pay more to receive a lift (the bidder/buyer) or wants to receive less for giving a lift (the seller).

Information on their usual commuting method (private/company car, bus, train, metro, bicycle, on foot); from this the level of impatience was generated.

To study which variables influenced the level of irritability caused by the traffic jams in “Mordor”, the collected survey data was analysed using data mining methods. The main factors influencing the level of irritability experienced by the commuters in this area were: age, travel time, and, to a much lesser degree, gender.

To optimize the parameters of the devised system, an in-depth analysis of the collected survey data was necessary to enable data enrichment and the “extraction” of the knowledge “hidden” in the data. The first stage of the study classified the answers to find out to which extent “Mordor” employees are willing to take part in gamification (take passengers or become passengers).

Out of 337 respondents, 45 were completely uninterested in carpooling—neither in being a potential passenger nor a driver. When analysing the remaining 292 surveys, a division into two classes/categories was made (in accordance with Jenks natural breaks classification method): “low” (a person mildly interested in carpooling) and “high” (potential participants in gamification).

**Figure 3:**
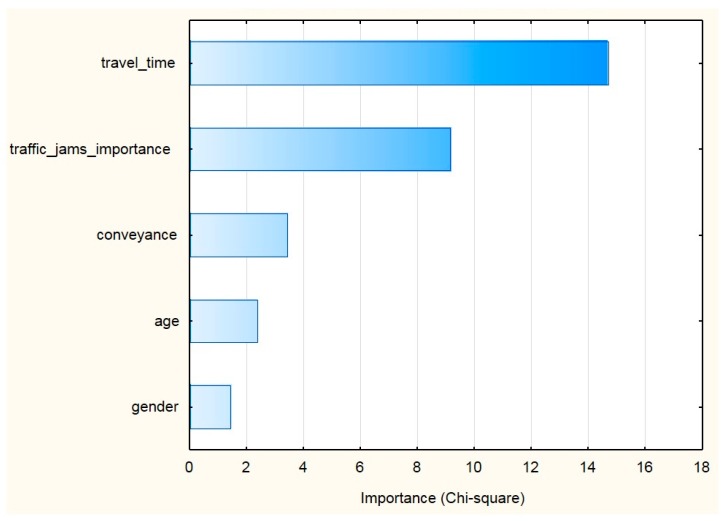
The ranking of predictors’ importance explaining the users’ inclination towards carpooling with gamification.

The most important variables influencing the results of the multivariate classification were: travel time; the degree of importance of the traffic jams problem to a person; conveyance; age; and, gender. (For ranking of predictors see Figure 3).

**Figure 4:**
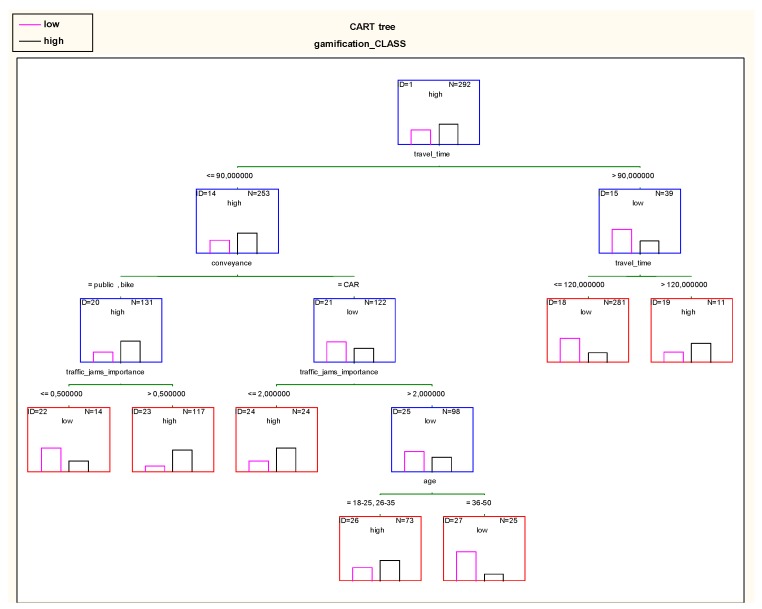
Classification and regression tree (CART) explaining the level of interest in carpooling and gamification.

Based on the five variables in Figure 3, a classification and regression tree (CART) explaining the issue was built (using reduced error pruning algorithm for the optimal size of the final tree). The travel time served as the main criterion of division (Figure 4). The results indicate that if a person travels more than 90 min (both ways), and then they are only slightly interested in carpooling. Fortunately, there were relatively few such respondents: 39 individuals when compared with 253 whose travel time is shorter than 90 min and who are potentially willing to use gamification and carpooling. Among those with travel times of less than 90 min, those travelling by car exhibit low interest, while those using public transport or bicycles exhibit high interest. Subsequent branches show how “irritability” caused by traffic jams and the age of respondents influence the decision.

The primary advantage of the system is that extracting rules is easy, e.g., for leaf No. 27 SELECT * FROM <TABLE> WHERE “travel_time” ≤ 90 (min.) and “conveyance” = ‘CAR’ And “traffic_jams_importance” > 2 And “age” = ‘36–50’.

Such results are useful when prototyping an expert system, in user segmentation, etc.

Once the binary classification was completed (“strong” or “slight” interest in carpooling and gamification), the next stage of research was to conduct further analysis by building a quantitative regression model displaying the degree of interest according to age, means of transport, and time of commute. The role of the CART model was to explain the respondents’ preferences using a two-class division: strong or mild interest in carpooling. The regression model is the next step of the analysis, which facilitates the modelling of this dependence as a continuous dependence. “Interest in carpooling and gamification” constitutes the dependent variable in this model (in a quantitative scale), while the independent variables are: age, means of transport, and time of commute.

There are many methods of building regression models that consider many variables. Radial basis function (RBF) networks were used in this study, to include variables expressed in different measuring scales (qualitative, quantitative, and ranking scales), and create neural networks with strongly nonlinear activation functions [69].

**Figure 5:**
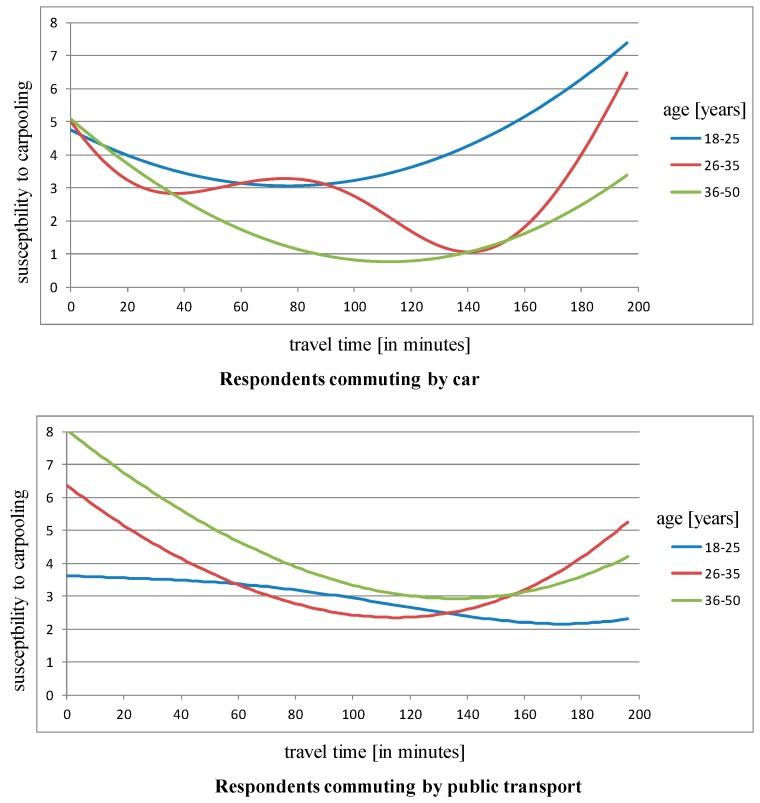
Inclination towards carpooling by means of transport (**upper:** car, **lower:** public transport), travel time, and age.

In Figure 5, the local extremum of interest for respondents aged 25–36 who commute by car appears when travel time is about 80 min. This enables an appropriate system parameterization and the development of a personalized “incentive” system dedicated to individuals of a certain age, gender, and who use particular means of transport.

### 4.2. Simulation of Gamification Mechanism

Following the definition that “gamification is the use of game elements and game-like thinking in non-gaming environment” [70], the authors of the article employ the gamification approach in a pretended reality, which is the reality seen on a smartphone or on a computer screen. Thanks to trust mechanisms (as described in [28]), authors obtain the realization of the results of the game in reality, that is the realization of the phenomenon of using a lift offered by other drivers. 

When analyzing the source data collected in the survey, the authors of this article pointed to the key factors that determine the inclination of the people working in this district of Warsaw towards gamification. Inspired by the name of this particular district of the city (the so-called “Varsovian Mordor”), the authors devised a prototype of an urban game that stimulates interest in carpooling and that draws upon J.R.R. Tolkien’s fantasy world. Game participants (called orcs) compete for “transport routes” by forging “alliances” through carpooling. The results of gamification are expressed in collecting “trophies”, namely points that place the user in the hierarchy. Apart from the individual rivalry of drivers and passengers, there is also a competition between “clans” (companies and institutions) in place, which results in winning a transferable (changing weekly) virtual title of the “Lord of Mordor”. What is more, the authors proposed using augmented reality (AR) to visualize the current status of particular “orcs” and “clans”. The authors of the article organized a demonstration of the prototype of the game during a gamification hackathon in the beginning of 2017. Because of the agreed conceptual assumptions of gamification and the adopted model of stimulating users, the authors are led to believe that implementing such an urban game would greatly increase the popularity of carpooling, and, consequently, would limit traffic jams, streamline road traffic, and reduce emission. Also, it would contribute to the development of social relations crucial in shaping a network geoinformation society in a smart city.

Due to the fact that full implementation of the proposed gamification model requires an estimation of the effects and expressing the results in a numerical form, to optimize the results the authors have attempted to simulate different variants of the game and various parameters of stimulating the users.

The game mechanisms we use are the following: competing for limited goods (that is the space in a car, with time and space limits), cooperating (that is creating collectives who plan they journey together), and the immediate feedback on the offer (the result is the acceptance or the rejection of the offer). The rules of the game are fixed and they convert agent parameters (such as: the price of the offer, kindness, capacity, region) into results.

To analyse which gamification mechanisms are going to produce the best result in the designed game, a simulation application has been created. The application has also been designed as a multi-agent system, as the target game is also meant to use agent-based programming paradigm.

Data enrichment and spatial data mining methods were used to develop dependency models that explain the inclination towards carpooling and gamification depending on several factors, including: the place of residence, age, travel time, means of transport, work hours, the need to pick up/drop off children at school, etc. Using the knowledge extracted from the source data, a multi-agent system was devised. It comprises 100,000 agents representing residents of particular districts of Warsaw who commute to “Mordor”. It was assumed that the distribution of characteristics in the population is analogous to the distribution of characteristics in the sample.

**Figure 6:**
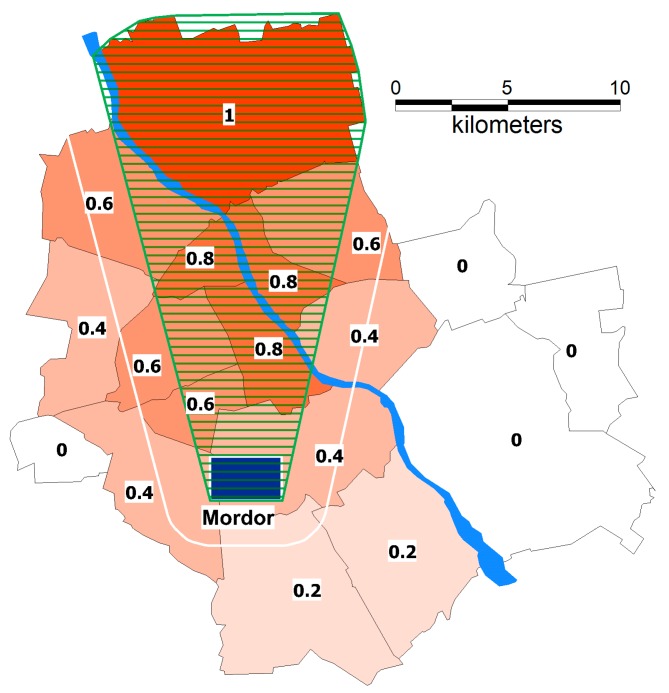
Weight assignment in a multi-agent system with spatial data enrichment and spatial analyses that use the convex hull (an example for a selected district).

It is assumed that the traffic problem in the Varsovian “Mordor” can be solved using a market-oriented multi-agent system. A day is divided into 1440 periods (minutes). In every period, multiple agents announce an auction in which they wish to sell their car ride. Every auction has determined regions, from and where to the driver travels. We define the |D| × |D| matrix (where |D| is the number of districts in Warsaw) that holds the probability of reaching the district while travelling to the driver’s home district. In Figure 6, the analysis of one of 16 districts of Warsaw is shown (an identical analysis has been performed for every district). A convex hull was created around the district and the area of “Mordor”, finding areas that lie within the hull, are adjoined or are nearby (a buffer of a specified radius), etc. If the value is equal to 0.0, it means that the district cannot be reached; a value of 1.0 means that it is always possible to reach the district. Each driver has a parameter called kindness, ranging from 0.0 to 1.0, the higher the kindness the more likely the driver goes off the set route. Such assumption reflects the agent’s decision on the route. By performing cartometric analyses, a specific kind of “data enrichment” was done. The “raw” data obtained through the survey were “enriched” by assignment to an appropriate spatial location and by properly classifying the respondents’ answers. In Figure 6, a method of weight assignment for just one district of Warsaw has been presented. It indicates that “the more on the way the passenger is from the driver’s place of residence to the driver’s place of work, the more inclined the owner of the car is towards carpooling”.

Presumably, a driver with 100% probability (1.00) takes passengers that are encountered directly on their way. This probability decreases with the potential necessity of a detour. In Figure 6, the analysis of one of 16 districts of Warsaw is shown (an identical analysis has been performed for every district). A convex hull was created around the district and the area of “Mordor”, finding areas that lie within the hull, are adjoined or are nearby (a buffer of a specified radius), etc.

Thus, the environment in each period deals with the multiple distributed sellers’ auctions of a specific good (target district), where the bidder can buy the travel to another district with specified probability (the goods are probable substitutes). Moreover, the number of goods to sell can be equal to 0, 1, 2, or 3 (unimodular goods auction). The auctions in subsequent periods are associated with each other, as the decisions made in the previous auction influences subsequent auctions. It is assumed that the solution is built iteratively (an arrow of time).

Thus, to summarize, the trade concerns multi-period, distributed, multiple, sellers’ auctions for probable substitutable unimodular goods.

The seller announcing the auction must specify:

- The threshold price.
- The source and target locations (one of which is always “Mordor”, and the other is always the driver’s home district).
- The duration of the auction (the maximum, from the moment of accepting the first passenger). The auction can end before the duration: if an agent reaches the end of the time boundary, or if they collect all the passengers.

### 4.3. Agents and Simulation

The simulation environment is designed and developed. The environment is designed using the multi-agent programming paradigm, as every person that travels to “Mordor”, and back is modelled as the agent (Figure 8). In this paradigm, an agent is a standalone piece of software that communicates with other agents, and, by making decisions, influences the total result of the whole system. The system is implemented in the FLAME (Flexible Large-scale Agent Modelling Framework) environment [71]. FLAME allows for a massive multi-agent system to run on multiple machines. Implementation assumes 100,000 communicating agents. An agent has the following properties:

- Impatience: values from 0 to 1, and specifies the level of willingness to take the car to work.
- Kindness: values from 0 to 1, this parameter specifies the level of the willingness of the driver to detour to drop the passengers to their place.
- Base price: specifies the base for the offer or the threshold price.
- Price modifier: used to modify the offer or the threshold price, depending on the time difference between the current moment and the default moment of departure.
- Capacity: of the car, this takes the integer values from 0 (when an agent does not want to take any passengers) to 3 or 5.
- Region: where the agent lives; in the simulation, these are Warsaw districts.
- Begin work: specifies the most anticipated time to start working.
- Begin dispersion: determines the acceptable deviation from the start of work. Together with “begin work” this determines the time boundaries for reaching the workplace.
- Finish work: specifies the anticipated time of finishing work.
- Finish dispersion: determines the acceptable deviation from the work finish. Together with “finish work” this determines the time boundaries for finishing the work.
- Allowable auction duration.

**Figure 7:**
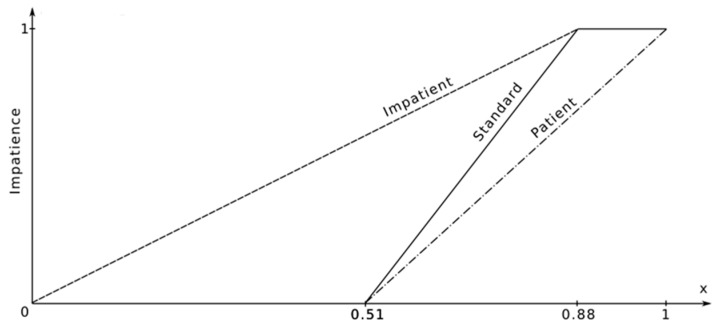
Generated data depending on the uniform distribution.

The following levels of impatience were assumed (Figure 7):

- Low impatience level.
- Standard impatience level.
- High impatience level.

With a probability equal to the percentage of the number of people travelling to work using public transport (51.7%), the impatience distribution is 0. It is because those travelling by means of public transport are natural candidates for passengers. Secondly, with a probability equal to the number of people travelling to work by car (11.7%), the impatience distribution is 1. Those using company cars are natural candidates for drivers—hosts of an auction. Finally, for those travelling by private cars (36.6%), we generate the “impatience” parameter from uniform continuous distribution ~U(0,1). The “kindness” parameter is generated on the basis of the results of the survey on the importance of the problem of traffic jams in “Mordor”. We interpret the results in the following manner: if the problem of traffic jams is very important to a person, this person will be more willing to use carpooling.

An agent has the following (viable) variables:

- State: specifies where the agent is now: (i) at home; (ii) travelling to work; (iii) in “Mordor”; (iv) travelling back home; or (v) back home.
- Want to travel: determines whether an agent wants to: (i) stay at work/home; (ii) attempt to travel; or, (iii) travel.
- Has car: specifies if an agent takes the car to travel (1), or if they want to travel as a passenger (0).

A developed agent (orc agent) can take one of five different roles, depending on the state of travel (passing the time), and on the decision of whether or not to take the car. The roles are:

- Idle: as the agent is either at work or home, an agent does nothing, and waits until they can begin the travel.
- Travelling with car: an agent announces the auction and collects co-travellers.
- Travelling without the car: an agent participates in auctions and tries to win one.
- Waiting for car: an agent who participated in the auction wins it and waits for the travel.
- Inviting to the car: an agent announcing the auction finishes it and invites the winners into the car.

**Figure 8:**
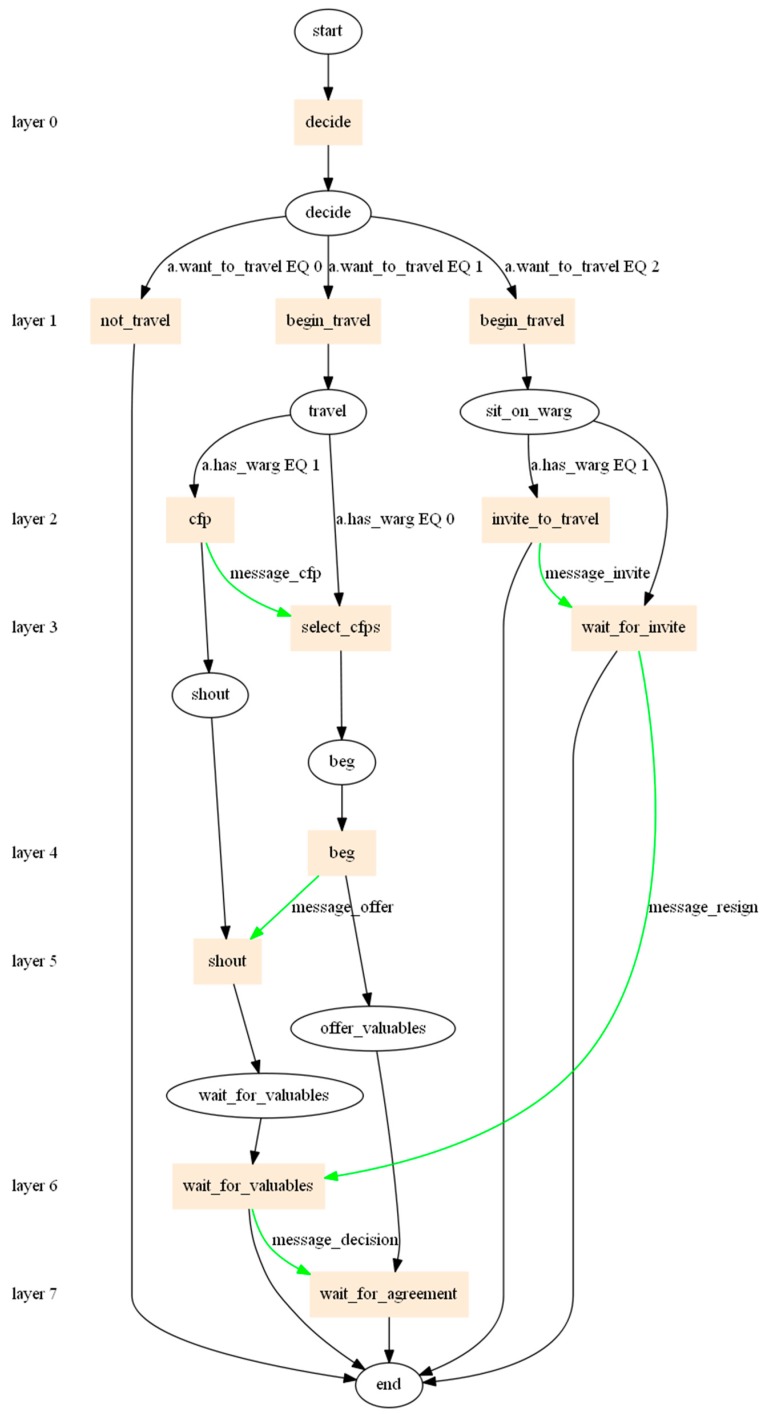
Flow of the agent’s actions. The flow represents five roles and the exchange of messages. Source: the authors’ own elaboration.

Figure 8 shows a model of the agent’s transitions in the designed and implemented simulation system. The scheme was generated using xparser application that is part of the FLAME framework in which the system had been implemented. The oval nodes represent the stages at which a given agent is in every round—a minute in a day. Their name, in accordance with the fantasy nomenclature, have the following meanings: start—the beginning of the round; decide—a decision on the path in which the algorithm of the agent’s actions will be continued; travel—the beginning of the travel process; shout—announcing the auction; beg—making offers; offer_valuables—if the agent has been chosen as the winner of the auction, she/he pays for the travel; wait_for_valuables—the agent who has submitted the auction is waiting for the payment from the passengers; sit_on_warg—agents are waiting to start their journey; and, end—the end of the round. Rectangular nodes represent actions—the implemented functions. Below is a description of the actions.

The model of agent’s transitions shall be analysed (See Figure 8, the flow of the agent’s actions; the flow represents five roles and the exchange of messages). This diagram illustrates the path of an agent during every minute of a day. The decide action—depending on the value of the variable state, the current time *t*, parameters signifying the beginning *t_beg_*/and the end tend of work, a delay in the beginning/end of work (*t_dispb_*/*t_dispe_*), and a random variable—in every quantum of time, the value of this variable for the state want_to_travel is determined. If an agent is at home (state = 0) and *t* < *t_beg_* − *t*_dispb_, and then an agent remains inactive. Otherwise, the value of a random variable with a uniform distribution is compared with the value *r* ≥ |(*t* − *t_beg_*)/*t_dispb_*|. Therefore, the closer an agent is to the beginning of work, the higher the chance that an agent will be likely to start the journey. It occurs analogously for the end of work (state = 2). On the other hand, when state equals 1 or 3, the value of the state variable want_to_travel equals 2.

Subsequently, a divergence occurs. It depends on the state variable want_to_travel; if want_to_travel = 0, then an agent remains inactive. If want_to_travel = 1, then an agent begins her/his efforts to begin the journey. If an agent is traveling by car (warg), she/he announces the auction. If an agent is a passenger, she/he seeks an auction and submits appropriate offers.

The path of an agent who begins her/his efforts to go home or to get to work shall be analysed. In the begin_travel action, a value of the state variable has_warg is established; it defines whether an agent is a driver who travels by her/his own car (has a warg in Mordor) or whether an agent is a passenger (does not have a warg in Mordor). This state variable depends on the impatience parameter and the current time t. When the impatience parameter equals 1, namely an agent is very impatient, then she/he always travels by her/his own car. When impatience = 0, then an agent is very patient and then she/he is always a passenger. For other values of this parameter (between 0 and 1), an agent first tries to be a passenger and to submit offers on the auctions that are already taking place; afterwards, having announced her/his own auction, an agent takes her/his own car and goes to work. This moment, for the travel to work, is determined by: *t_beg_* − *t*_dispb_ * (1 − 2 * impatience).

Depending on the value of the state variable has_warg, an agent follows one of the paths: an auction’s operator when she/he is a driver or a participant in an auction when she/he is a passenger. A driver announces the auction (message_cfp), meaning that she/he wants to take passengers, and she/he announces where from and where to she/he travels. In every quantum of time, a passenger collects announcements and responds to those that are consistent with her/his location or destination (message_offer). The offer price for a place in a car increases with time, which means that an agent seeks more attractive offers in the beginning but, as time passes, is ready to pay more when she/he does not win. A driver receives offers, checks whether the offer prices are appropriately high (the threshold price parameter, tp), and accepts the offers with the highest prices. Subsequently, a driver responds favourably or adversely (message_decision) to the offer. A driver holds an auction until the car is full or until the auction process is over (so that the first approved bidder does not have to wait too long).

A passenger who receives a positive response passes to phase want_to_travel = 2. She/he waits for the beginning of the travel, that is when a driver informs her/his that the travel begins (message_invite). If the wait is too long, she/he sends a message to the driver that she/he resigns (message_resign), and she/he travels using public transport instead. If a passenger/agent has been waiting to be accepted for a given time, and then she/he also travels using public transport. When a driver/agent decides to travel, then the agents who are her/his passengers change their statuses to traveling (work/home).

The simulation covers one day, divided onto 1440 units (24 h × 60 min). Every minute, an agent can decide: (i) whether they want to begin the travel (to work or back home), and (ii) how to travel (take their own, travel with another agent, or use public transport).

The travel method decision depends on the level of impatience of an agent:

- If the impatience is equal to 0 (an agent is utterly patient), they never take the car to work, and participate in the auctions as a bidder. If the agent is not able to win an auction up to the end of the time boundary, they travel using public transport.
- If the impatience is equal to 1 (an agent is utterly impatient), they always take the car to work, and always announce an auction (they are the seller). They announce the auction every 1 min, until they collect the co-travellers, or reach the time boundary.
- Finally, if the impatience is between 0 and 1 (an agent is moderately impatient), every time (until they collect at least one companion) the agent draws a random number *r* from the uniform distribution, and compares the value to the ratio of time *t* passed from the moment of the possible beginning of the travel (*t_beg_* − *t_disp_*) to the dispersion of the travel beginning 2 * *t_disp_* (see Equation (1)). The probability of deciding that the agent should begin the travel rises with the time passed, and is equal to 0.5 for the *t_beg_* moment. With the passage of time, it is more likely for an agent to take a car and announce the auction, while in the beginning they are more likely to be a bidder.

(1) $$r>\frac{t-(t_{\mathrm{brg}}-t_{\mathrm{disp}})}{2*t_{\mathrm{disp}}}$$

### 4.4. Environment

The environment has different running options:

- The number of agents.
- Random seed.
- Relationship of the threshold price and the number of passengers. Three relationships are assumed:
  o The threshold price does not depend on the number of passengers (constant).
  o Exponential dependence: *tp* = *bp*/(1 + *passengers*).
  o Linear dependence: tp = bp * (1 − c * passengers) (*c* = 10%).

### 4.5. Parameters Essential for the Gamification Mechanism

Data mining methods (de facto spatial data mining) were applied to the exploratory analysis of data collected in the survey. This enabled the construction of a generalized regression model that gives a broad overview of how particular factors (e.g., commute time, age, gender, means of transport, etc.) influence the inclination of the people working in this district of Warsaw towards carpooling and gamification. The parameters that are particularly essential for the gamification mechanism are: impatience, kindness, allowable duration of the auction, and the dependency of price on the number of passengers. Therefore, the authors have proposed a number of tests to examine their influence on the simulation.

## 5. Experimental Results and Discussion

**Figure 9:**
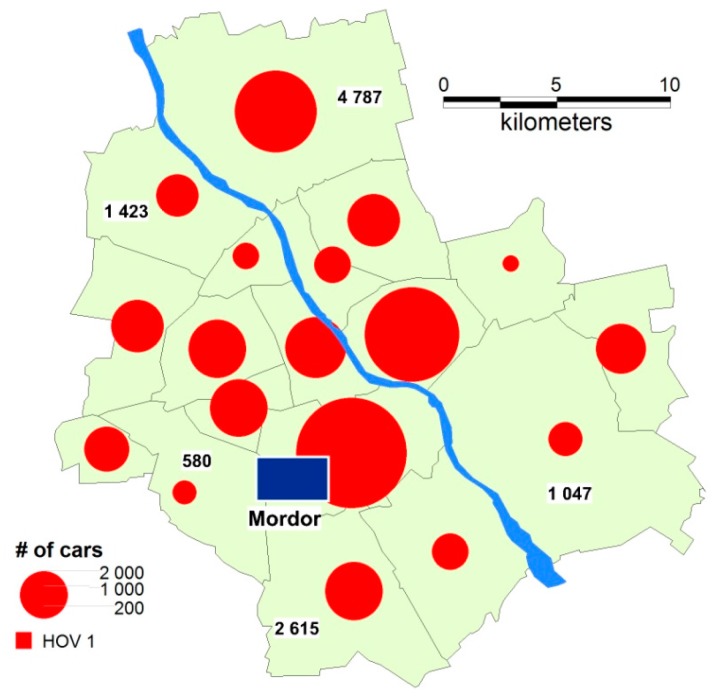
Reference data: the number of cars leaving particular districts of Warsaw for “Mordor” (every car is occupied solely by the driver).

The research revealed strong disparities in the obtained results, depending on the simulation parameters (Table 1). The state (modelled on the real one) in which every day around 44,000 cars leave districts of Warsaw for “Mordor” and come back home during the hours specified by studying the representative sample served as the reference data. In the reference data, it was assumed that every vehicle is occupied by one person (the driver), resulting in the high occupancy vehicle (HOV) parameter for HOV2 = 0, for HOV3 = 0, HOVover3 = 0 (Figure 9). The remaining 56,000 people working in “Mordor” commute using bicycles or public transport. Their inclination (similarly to that of the drivers) towards gamification and carpooling was set based on the survey and analyses. Linearly scaled diagrams are shown in the thematic map: red circles indicate the number of drivers—and cars at the same time—that leave a particular district for “Mordor” and come back in the evening.

**Figure 10:**
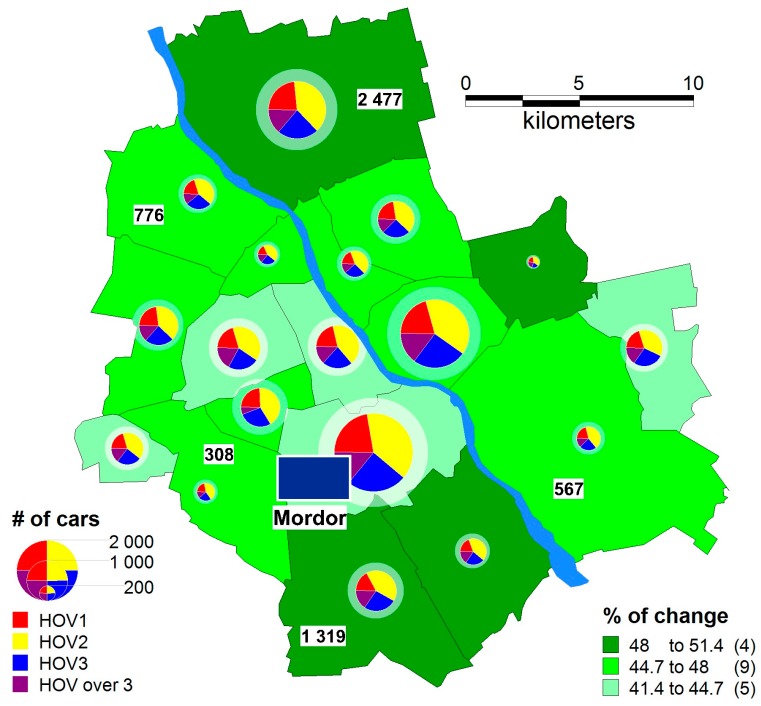
The number of cars (the number of passengers): a pie chart and the percentage change in the number of cars compared to the referential variation—a reference auction.

The results of the simulation incorporating the usage of a gamification-based carpooling model are extremely interesting. Even the simplest type of auction results in reducing the number of cars by half; where two passengers travel in 40% of cars, three passengers in 24%, and more than three passengers in 14% of cars. A spatial distribution for this model is shown in a thematic map (Figure 10), where the size of the pie chart indicates the number of cars leaving a particular district for “Mordor” (in the background, a semi-transparent chart for reference data is displayed for comparison). The chart is divided into colours, indicating how many cars with one, two, three, and more than three passengers there are. The intensity of the green colour indicates the percentage change of the number of cars in a particular district. In the devised model, a change is beneficial and always increasing; therefore, the darker it is, the better.

The analysis of the results obtained (Table 1) indicates that the key parameter influencing the number of passengers commuting to work together (HOV) is not just the cost of carpooling, but also the “patience” of agents. For “impatient” agents, the number of cars, relative to the reference variation, is reduced by one-third; for “patient” agents, it is almost two-thirds.

**Figure 11:**
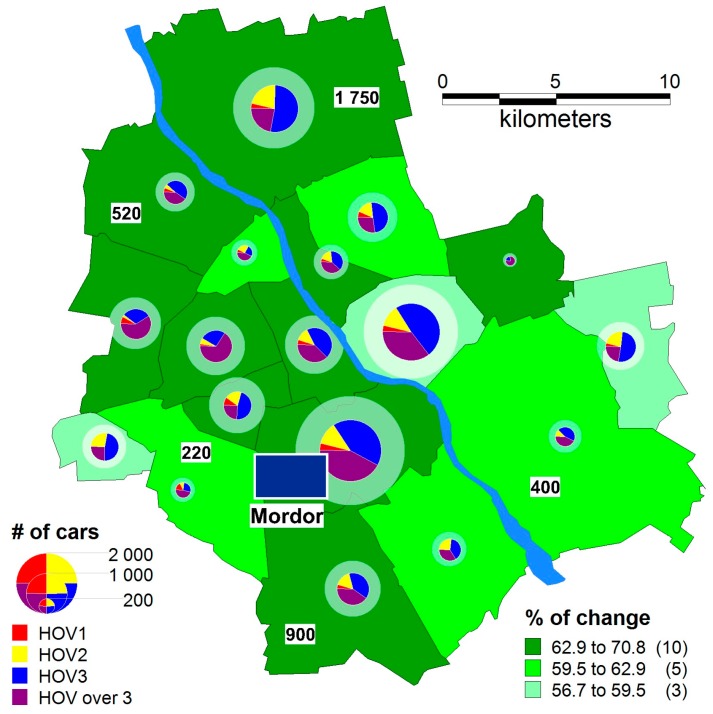
The number of cars (the number of passengers): a pie chart and the percentage change in the number of cars compared to the referential variation—“patient agents”, the price is decreasing exponentially.

For the “patient agents” variation, the price decreases exponentially (Figure 11), the number of cars decreases by almost two-thirds, HOV is just 4% (on average in districts), and an overwhelming majority (80%) are HOV3 or HOVover3.

**Figure 12:**
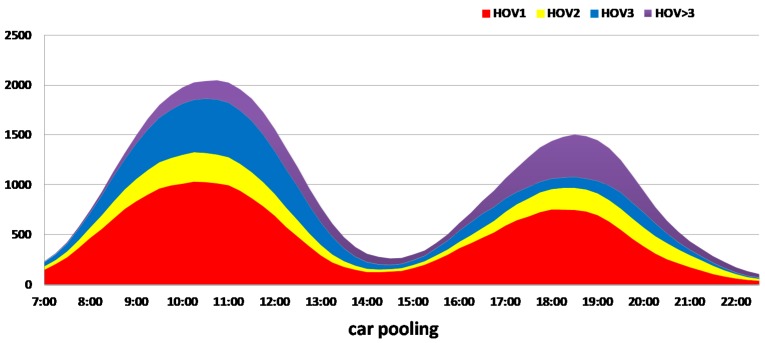
The change over time: the number of cars and passengers, for the reference variation and for the “patient agents, the price is decreasing exponentially” variation.

The analysis of the distribution of changes over time, which shows how the number of cars changes during particular hours for the reference and auction versions, is shown in Figure 12. For the “patient agents, the price is decreasing exponentially” variation, the changes in the number of cars and passengers according to the time are shown on a cumulative graph.

The authors have also considered the practicality of the potential implementation of the devised model. The cost of developing, testing, and parameterizing a mobile application that facilitates carpooling using gamification is relatively small. Developing an appropriate business model that enables all the participants of the process to benefit economically, estimating the impact of the proposed solution on traffic jams in the city, and reducing air pollution are all crucial. For approximate calculations, it was assumed that an average length of the route is 20 km both ways, and that the average fuel consumption in traffic jams is about 8 L per 100 km. For a gamification model that facilitates a decrease in the number of cars by 20,000 a day, it gives a total CO_2_ emission of 140 tonnes a day. In a year, the emission is 34,000 tonnes for gasoline vehicles. Assuming that half of the cars are diesel vehicles, this value increases to almost 50,000 tons of CO_2_ a year. Therefore, the proposed solution not only saves time and energy, but it also reduces CO_2_ emission, and, thus, air pollution.

### Simulation of Changing the Speed of Vehicular Traffic in “Mordor”

**Figure 13:**
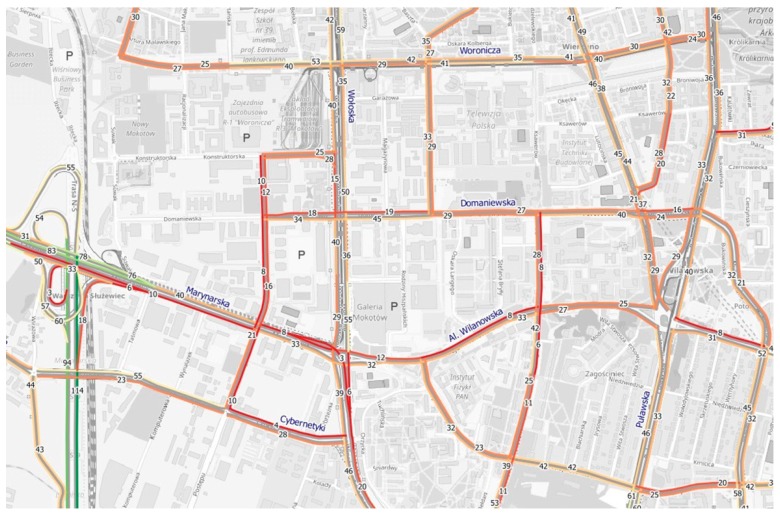
Average speed on the streets of “Mordor” during rush hours (based on monitoring data provided by CE-Traffic, a.s.).

Using the reference data and a model developed in cooperation with CE-Traffic, a.s. company, a map of traffic intensity in “Mordor” during rush hours (Thursday, 6 p.m.) was devised. This map (Figure 13) shows data from fleet vehicles monitored by CE-Traffic, a.s. Significant traffic difficulties (speed of a dozen or even a few kilometres per hour) are presented. To verify the average speed on streets in “Mordor”, investigations were made using an application programming interface (API) and software development kit (SDK) from HERE Maps and traffic speed data taken at 15-min intervals during the day, as downloaded from HERE [72].

**Figure 14:**
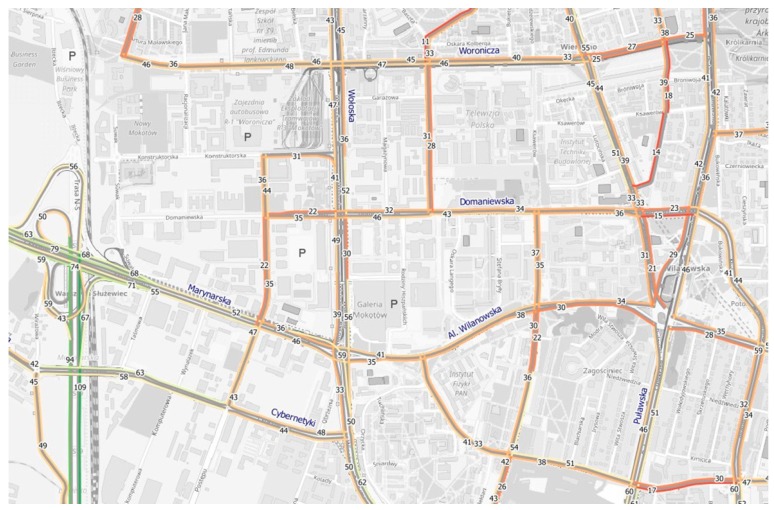
Average speed on the streets of “Mordor” during rush hours (based on simulation results for the variation of an auction with “patient” agents).

The next map (Figure 14) shows the results of speed estimation during rush hours when the developed model is implemented. The potential speed values were calculated based on the estimated number of cars, and the class and width of the road by using the so-called fundamental diagram of traffic flow.

**Figure 15:**
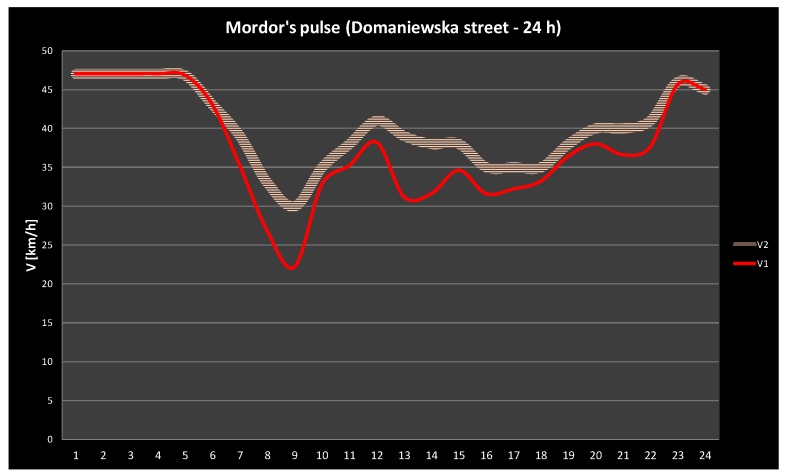
A daily change in speed for Domaniewska Street—reference data and the auction with “patient agents” variation.

Using the developed model and the methodology of determining speed based on the estimated number of cars, the class, and width of the road, and similar parameters, on a selected section of a road (a section of Domaniewska street), a 24-h change in speed (in km/h) was compared. A 24-h change in speed is shown in Figure 15: the fleet monitoring is represented in red; pink signifies the estimated speed model. The minimum speed expected in the model is about 30 km/h, ensuring the traffic flow (Figure 15).

## 6. Conclusions

This research demonstrates that using appropriately stimulated gamification, geoinformation technology and decision support systems can significantly impact the popularization of carpooling. Dramatically reducing the number of cars (in some models by as much as 65%), while maintaining the same number of commuters may not only significantly contribute to alleviating a city’s transport problems without serious infrastructural changes (parking lots, widening the streets, etc.), but may also significantly reduce energy consumption. It is equally important to incentivize social activity by skilfully stimulating the residents’ cooperation through gamification techniques. The authors of this article suggest that these types of social innovation actions supported by IT solutions will prove crucial for the functioning of smart cities in the IoT development era. Modern technologies are not the decisive elements of building a truly smart city. The most important aspect in building a smart city is including its inhabitants in the process of building a sustainable and comfortable urban space.

Smart cities are not just an idea. They are, ultimately, the process of skilfully using information resources that are associated with each sphere of a city’s activity. Smart cities should promote tools that support sustainable ecological and social growth, as well as actions related to social participation and civic engagement. It is crucial to encourage residents, who comprise the open geoinformation society, to voice their own opinions on the city and their vision for its transformation. New generation geoinformation tools in combination with gamification techniques may facilitate the process of gathering data. Most importantly, however, they make it possible to convert the data into useful information—enabling spatial knowledge acquisition, and, consequently, using that knowledge to create innovative and socially useful decision support models. The authors of this article propose such an approach—using the methodology of gamification, multi-agent systems, and appropriately processed and enriched data—to contribute to the popularization of carpooling amongst the residents of smart cities.

What should be emphasized is that this solution is not restricted to the area of the “Mordor of Warsaw”. At the core of this approach is the development of a methodology—smart city specific—that reduces energy emissions and optimizes the traffic flow by using gamification to stimulate carpooling. Obviously, the system parameterization requires the analysis of each city’s survey data by means of data mining techniques, but the general framework remains the same.

The effective stimulation of residents by using an aptly constructed gamification system and urban games (“serious game” type) is an incredibly interesting idea, including from a scientific standpoint. However, since this idea mostly deals with applied social sciences, it will be the subject of a separate publication.

## Figures and Tables

**Table 1:** Results of simulation experiments.

No.	Variation Name	Number of Cars	L1/L0 (%)	HOV1 (0%)	HOV2 (%)	HOV3 (%)	HOVover3 (%)
**1**	Referential	43,855	100	43,855	0	0	0
**2**	Auction, constant price	23,854	54.4%	5156	9487	5831	3380
				22%	40%	24%	14%
**3**	Price decreases exponentially	22,330	50.9%	6130	3690	8030	4480
				27%	17%	36%	20%
**4**	Price decreases proportionally	23,280	53.1%	5860	7100	5990	4330
				25%	30%	26%	19%
**5**	Impatient agents, price decreases exponentially	28,850	65.8%	7810	14510	6090	440
				27%	50%	22%	1%
**6**	Impatient agents, price decreases proportionally	29,830	68.0%	8090	16720	4590	430
				27%	56%	16%	1%
**7**	Patient agents, price decreases exponentially	16,290	37.1%	690	2490	7040	6070
				4%	15%	43%	38%
**8**	Patient agents, price decreases proportionally	17,490	39.9%	660	5790	5670	5370
				4%	33%	32%	31%

## Appendix A. Survey Questions

The research is conducted by the researchers from the Warsaw University of Technology. The goal of the research is to test the concept of using gamification in the implementation of the “smart city” idea.

1. As far as the problem of traffic jams in “Mordor”—how important is it to you? (scale 0–100)
2. At what time (hour) do you usually start work?
3. At what time (hour) do you usually finish work?
4. On average, how much time (in minutes) does it take you to go to/back from work? (one way)
5. How much room for manoeuvre do you have when it comes to changing the time at which you start/finish work (in minutes)?
6. In which district of Warsaw do you live? (one of 17 districts or “outside of Warsaw”)
7. In which part of “Mordor” do you work? (address format: street, building no.)
8. Which means of transport do you mostly use when commuting to work? (private car, company car, underground railway (metro), tram, bus, commuter rail, bike, on foot, other)
9. How much time does it take you (in minutes) to go through “Mordor” to get to one of the outbound highways?
10. Do you have to go somewhere before/after work? (take children to/from school or preschool, shopping, giving a partner/a friend a lift, other)
11. How would a perfect work commute look like?
12. What could make you want to share YOUR vehicle with other workers? (NOTHING, money, bonus points, free lunch, more time off, my boss’s satisfaction, other)
13. Have you ever shared a vehicle when travelling to/from work? (Y/N)
14. What could make you want to share SOMEBODY’S vehicle when travelling to work? (NOTHING, money, bonus points, a pleasant driver, talking about business in the car, social contacts, other)
15. Evaluate the place (area) in which you work in “Mordor” as far as the ACCESSIBILITY OF TRANSPORT (scale 0–100)
16. Gender (F/M)
17. Job position
18. Age (18–25, 26–35, 36–50, >51)
